# Characterization of Adult Patients With Neurometabolic Disorders: A Cross‐Sectional Study at a Tertiary Neurology Center in Sweden

**DOI:** 10.1002/jmd2.70115

**Published:** 2026-08-01

**Authors:** Boel Ernerdahl, Ashraf Yahia, Andreas Puschmann

**Affiliations:** ^1^ Division of Neurology, Department of Clinical Sciences Lund Lund University Lund Sweden; ^2^ Department of Neurology Skåne University Hospital Lund Sweden; ^3^ SciLifeLab, Lund University Lund Sweden

**Keywords:** adults, International Classification of Inherited Metabolic Disorders, neurometabolic diseases, phenotype, precision medicine, social support

## Abstract

Adult patients with inherited metabolic diseases are often overlooked. Limited data on this population hinder adequate planning of their clinical and social care. In this retrospective, observational, cross‐sectional service evaluation study, we reviewed the electronic medical records of adult patients with inherited neurometabolic diseases (NIMD) managed at Skåne University Hospital between 2020 and 2024. Skåne University Hospital, located in Region Skåne in Southern Sweden, is one of three specialized tertiary centers for inherited metabolic diseases in Sweden. The aim of this study was to characterize these patients demographically and clinically and to describe their treatment profiles, functional status, and social support. We identified 59 patients. Most patients had disorders of intermediary energy metabolism (21/59; 35.6%) or lipid metabolism (14/59; 23.7%), including 10 patients (16.9%) with adrenoleukodystrophy. Disease‐specific treatments were provided to 65.4% of patients, and 83.6% were under continuous follow‐up. Sixteen patients (27.1%) were fully independent, lived in their own homes, did not require societal support nor mobility aid, and worked/studied full time/retired due to old age. More than half of the patients (50.8%) received no disability or social support. In conclusion, adult patients with NIMD represent a heterogeneous group with variable clinical and social needs. Despite the rarity of individual NIMDs, disease‐specific treatments were commonly used in our center. Further studies, particularly long‐term follow‐up studies, are required to better understand NIMD clinical trajectories and to optimize both medical management and social support for adults with NIMD.

## Highlights

- Most adult patients with inherited neurometabolic diseases managed at the Department of Neurology, Skåne University Hospital, Region Skåne, Sweden, between 2020 and 2024 had disorders of energy or lipid metabolism and received disease‐specific treatment.
- About half received no disability or social support, with more than one‐quarter requiring none.
- Medical and societal support needs varied widely among patients.

## 1 Introduction

The number of adults living with inherited metabolic diseases (IMD), including neurometabolic diseases, continues to increase [1]. This increase is not only due to improved survival of children with IMD but also due to increased diagnosis in adulthood [2, 3]. A recent multicentric study reported that 40% out of > 10 000 patients included were diagnosed in adulthood [1].

IMD manifestations in adults can differ markedly from those in children, possibly due to differences in the relative importance of metabolic pathways and substrates, the long‐term accumulation of toxic metabolites, exposure to challenging metabolic and physiological states such as fasting and pregnancy, and organelles' down‐performance due to aging among others [4]. Most adults with IMD show neurological and/or psychiatric manifestations [5]. This is likely due to progressive impairment in pathways implicated in neurodegeneration and neuroinflammation [6, 7]. Herein, we refer to those IMD forms with neurological manifestations as inherited neurometabolic diseases (NIMD). Thus, IMD includes disorders with and without neurological manifestations, while NIMD only includes disorders with neurological manifestations.

The difference in IMD manifestations between adults and children complicates their clinical diagnosis. The amount of literature and resources, including trained personnel, dedicated to IMD in adults is still lagging behind the situation in pediatrics [2, 5, 8]. Furthermore, parents and siblings are not often available for trio sequencing and segregation analysis, complicating genetic diagnostics [5].

Beyond diagnosis, adults with IMD have their peculiar social and psychological needs that differ from the family‐centered children's needs. For example, aspects such as independence, relationships, and reproduction become more prominent after adulthood [2, 9]. A robust social and community support is essential for integrating adults with IMD in society and enabling them to fulfill their potential, especially since many have neurobehavioral impairments [10]. Data collected from multiple European countries showed a widespread lack of social support for IMD patients in Europe, with some differences between countries [11].

In Sweden, 21 healthcare regions provide most primary, secondary, and tertiary care [12]. In 2024, three centers for national highly specialized care in IMD were assigned in Sweden, namely Skåne University Hospital, Region Skåne, Southern Sweden, Sahlgrenska University Hospital, Västra Götaland, Western Sweden, and Karolinska University Hospital, Stockholm, Central Sweden. The Department of Neurology at Skåne University Hospital provides care for adults with NMID. The aim of this study was to describe the demographic and clinical characteristics, treatment profiles, social support, and functional status of adults with NMID seen at the Department of Neurology, Skåne University Hospital, to support improving clinical management and care planning for this group of patients.

## 2 Methods

This was an observational cross‐sectional service evaluation study conducted in the Department of Neurology, Skåne University Hospital; and included patients seen between 2020 and 2024. An outline of the NIMD care provided at the Department is in the Supporting Information. Data was collected retrospectively from Region Skåne's electronic medical record (EMR) system. Patients were identified using two strategies. First, EMR was searched using relevant International Classification of Diseases Tenth Revision (ICD‐10) codes. The codes used are provided in Table S1. In the second strategy, neurologists at the hospital provided a list of adult patients with NIMD. Both lists were reassessed to exclude misclassification and overlap. Inclusion criteria were: confirmed neurometabolic diagnosis (biochemically and/or genetically), age ≥ 18 years, contact with Skåne University Hospital between 2020 and 2024, and survival at the time of review. Diseases were classified as metabolic if they were included in the International Classification of Inherited Metabolic Disorders (ICIMD) [13]. Patients' EMR were manually reviewed for those inclusion criteria. Figure S1 summarizes our patient identification strategy and its outcome. Data was collected by manual review of EMRs between February and March 2025. Extracted variables included: ICD code, diagnosis, sex, age, age at onset and diagnosis, symptoms (Human Phenotype Ontology terms), genotype, treatment (disease‐specific and symptomatic), follow‐up type at the department (continuous vs. one‐time), living arrangements, social support, mobility aids, and employment. We regarded dietary measures, formulas, vitamins, cofactors, chelating agents, stem cell transplantation (restricted solely to patients with adrenoleukodystrophy), and orphan drugs as disease‐specific treatments. Swedish social benefit terms were translated and explained (Table S2). Data was summarized using descriptive statistics. Metabolic diseases were hierarchically classified according to the ICIMD [13].

## 3 Results

We retrieved and studied 59 patients with a diagnosis code of neurometabolic disorders. Females constituted 57.6% (34/59) of our final study population. Out of 55 patients with available age at onset, 30 (54.5%) had their disease start before 18 years. Approximately 32% (18/56) received a diagnosis before 18 years of age. Table 1 summarizes the patients' demographic data and Figure S2 shows the distributions of ages at onset, diagnosis, and most recent contact with the Department of Neurology. Patients diagnosed in recent years had longer intervals between symptom onset and receiving a genetic diagnosis, and many received their genetic diagnosis as adults (Figure S3). Most patients had ongoing follow‐up at the Department of Neurology, whereas 9 (16.4%) only had a single consultation. Of those 9 patients, 3 relocated to another region/country, one was lost to follow‐up, and the rest were referred to primary or secondary care centers.

**TABLE 1:** Patients' ages and management.

Group	Age at onset^a^	Age at diagnosis^a^	Age at most recent contact^a^	Disease‐specific treatment^b^	Symptomatic treatment^b^	Continuous follow‐up^b^, ^c^
Females	*n* = 31; 21.1 ± 23; 11 (0–69) years	*n* = 31; 28.9 ± 22.1; 29 (0–71) years	*n* = 34; 42.4 ± 18.9; 38 (19–77) years	64.5% (20/31)	67.7% (21/31)	87.5% (28/32)
Males	*n* = 24; 18.1 ± 16.6; 18.5 (0–55) years	*n* = 25; 29 ± 17.6; 30 (0–57) years	*n* = 25; 40.2 ± 13.9; 39 (19–67) years	66.7% (16/24)	79.2% (19/24)	78.3% (18/23)
Total	*n* = 55; 19.8 ± 20.4; 14 (0–69) years	*n* = 56; 29 ± 20; 29.5 (0–71) years	*n* = 59; 41.4 ± 16.8; 39 (19–77) years	65.4% (36/55)	72.7% (40/55)	83.6% (46/55)

*Note:* Ages at disease onset, diagnosis, and most recent contact with our clinic are shown. The number of patients on disease‐specific treatments (such as special diet or enzyme replacement therapy) and symptomatic treatments (such as anti‐seizure drug or botulinum toxin) were shown as well as the number of patients on continuous follow‐up.

^a^ Presented as number of individuals depicted as *n*; mean ± standard deviation; median (range).

^b^ Presented as percentage (number of individuals/total number of individuals with available information).

^c^ Patients who had been followed longitudinally at our clinic, as opposed to patients seen only once.

**FIGURE 1:**
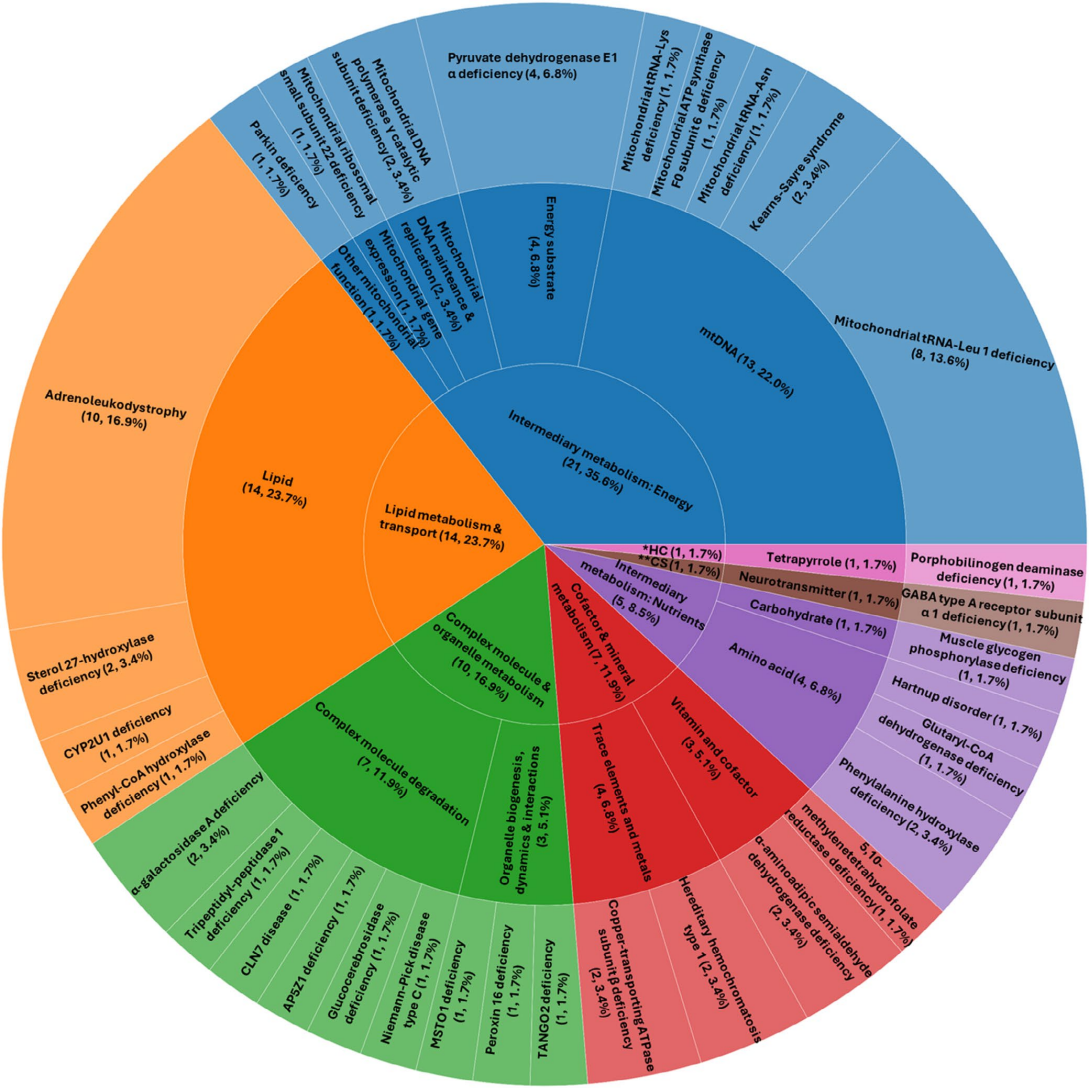
Categories of neurometabolic disorders identified. The metabolic categories of the detected diseases are shown. We classified diseases according to the International Classification of Inherited Metabolic Disorders (ICIMD) classification [13]. CS, cell signaling; HC, heterocyclic compounds.

The study population included 32 distinct NIMD (Figure 1 and Table S3). The most prevalent were adrenoleukodystrophy (OMIM 300100), identified in 6 females and 4 males, and mitochondrial tRNA‐Leu‐related disorder, identified in 6 females and 2 males. The most common major classes of metabolic disorders were disorders of intermediary energy metabolism (35.6%) and disorders of lipid metabolism (23.7%). X‐linked inheritance was reported in 16 patients (28.6%, 6 males and 10 females) and mitochondrial inheritance in 14 patients (25%). Fourteen patients (25%) had compound heterozygous inheritance, 10 (17.9%) had recessive, and 2 (3.6%) had dominant/de novo patterns.

Problems of vision, gastrointestinal and urinary systems, and musculature; abnormal movements; developmental delays; and psychiatric features were the most common manifestations (Figure S4). Four patients with adrenoleukodystrophy (3 females and 1 male) also had autoimmune diseases (rheumatoid arthritis, type 1 diabetes mellitus, psoriasis, and autoimmune thyroiditis, each in one patient); one also had adrenal insufficiency. One additional patient with adrenoleukodystrophy had adrenal insufficiency and no autoimmune disease.

Disease‐specific treatments were provided to 65.4% (36/55) of the patients, including dietary interventions and enzyme replacement. Approximately 72% received symptomatic treatments (such as antiepileptics) (Table 1). Six patients (10.9%) received no treatment.

Half of the cohort (30/59; 50.8%) received no formal societal benefits. About a quarter (16/59; 27.1%) met our strict, multifactorial composite definition of full independence (which additionally required full‐time working/studying or retiring due to age, living in one's own home, and using no mobility aids). Table 2 summarizes the patients' independence levels and the social support they were receiving.

**TABLE 2:** Elements of independence and social support.

Social characteristic	Number and percentage of patients
Living arrangements	– Living in their own home: 42 (71.2%) – Living with family/relatives: 15 (25.4%) – Supported living^a^: 1 (1.7%) – Nursing home: 1 (1.7%)
Disability or sickness support from society^b^	– No reported support: 30 (50.8%) – Personal assistance: 13 (22%) – Domestic services (including social care alarm): 2 (3.4%) – Any sickness benefit, long term and short term (Sickness compensation, activity compensation, sickness benefit): 9 (15.2%) – Legal guardian: 6 (10.2%) – School for Pupils with Intellectual Disabilities: 1 (1.7%) – Accompanying person and housing support: 3 (5.1%) – Daily work like activities: 1 (1.7%) – Accommodation with extensive support (accommodation according to Support and Service for Persons with Certain Functional Impairments and nursing home): 2 (3.4%)
Mobility aid^b^	– No mobility aid: 40 (67.8%) – Wheelchair: 14 (23.7%) – Rollator: 4 (6.8%) – Crutches: 3 (5.1%)
Work, studies, retired due to old age^c^	– Work to any extent: 21 (35.6%) – Studies: 5 (8.5%) – Retired due to old age: 5 (8.5%) – Not working/not studying/not retired due to old age: 27 (45.8%) – Not documented: 2 (3.4%)

^a^ Supported by the government according to the Act concerning Support and Service for Persons with Certain Functional Impairments.

^b^ Adds up to more than 100% as one individual can have more than one type of support and mobility aid.

^c^ One individual both worked and studied.

## 4 Discussion

We retrospectively studied 59 consecutive patients with NIMD from an adult neurology clinic at a national IMD center. We found a wide variability in their clinical symptoms, functional status, need for specific treatment and follow‐up, and of the societal support they received. IMD are generally more severe but also more well‐characterized in children than in adults, however, IMD may manifest at any age; patients with a very late age at onset, reaching 80 years, have been reported before [5].

Herein, we showed a broad age at onset that ranged from birth to 69 years old (Table 1 and Figure S2). The mean age at diagnosis in our study lagged ~10 years (median ~15 years) behind the age at onset (means were 29 and 19.8 and medians were 29.5 and 14 years, respectively). An increasing number of adult patients received genetically verified diagnosis in recent years, many of them a long time after symptom onset (Figure S3). This is attributable to improved accessibility and yield of genetic diagnostic tools rather than a true increase in time to diagnosis.

Adults with IMD have their peculiar phenotype but also support needs [2]. Most adults with IMD have neurological manifestations [5]. As we only studied patients at a neurology clinic, all had neurological symptoms. However, we also detected non‐neurological features such as arrhythmias and skeletal deformities (Figure S4). Non‐neurological features are well‐known in NIMD as these tend to afflict multiple systems [5]. Most patients in our study had disorders in intermediary energy metabolism and lipid metabolism. This high proportion was mainly driven by the high numbers of patients with adrenoleukodystrophy (16.9%) and mitochondrial diseases (35.6%). In a recent multicenter study on adult patients at centers for IMD, phenylalanine hydroxylase deficiency (phenylketonuria; 19.7%), alpha‐galactosidase A deficiency (Fabry's disease; 8.4%), adrenoleukodystrophy (3.9%), and various mitochondrial disorders (12%) were the most common disease entities; however, the authors acknowledged that those disease frequencies were biased by the relative expertise and historical interests of each center, and the relatively small number of centers [1]. In our study, the disease frequencies were also biased by the low sample size. We detected phenylketonuria in two siblings born outside Sweden, who had developed neurological symptoms. One study found a 5.1% incidence of adulthood neurological complications among patients with phenylketonuria in France; delays in initiating low phenylalanine diets and poor metabolic control during periods of critical neurological development in childhood increase the risk of such complications [14]. We also detected two patients (a male and a female) with Fabry's disease. Fabry's disease's incidence in Sweden was estimated to be 1.11/100000 [15]. This incidence estimate is comparable to estimates elsewhere [16]. Patients with known Fabry disease who receive enzyme replacement therapy are by convention treated at another health care unit, outside our clinic. On the other hand, glucocerebrosidase deficiency (Gaucher's disease) has a higher incidence in Sweden, 2.11/100000, due to a founder mutation in Northern Sweden that causes type 3 Gaucher's disease [15, 17]. We identified a single patient with (neuropathic) Gaucher's disease in our study who had contact with our neurology clinic and this patient did not originate from Northern Sweden.

Most patients presented with ocular or muscular signs and symptoms, and/or developmental and/or psychiatric features. This was in line with mitochondrial diseases being the most common disease entity in our study [18]. Four of the adrenoleukodystrophy patients we reported had autoimmune diseases. Screening for autoimmune diseases is not part of the management of adrenoleukodystrophy [19] as there is no established association between the two. Scattered case descriptions reported autoimmune diseases in patients with adrenoleukodystrophy; these included vitiligo and ulcerative colitis [20], Guillain‐Barré syndrome [21], type 1 diabetes mellitus [22], and multiple autoimmune disorders [23, 24]. Interestingly, all those reports described males with adrenoleukodystrophy and autoimmune diseases. We herein reported an additional one male and three females with autoimmune diseases. It remains unclear whether the association between adrenoleukodystrophy and autoimmune diseases was a true association or due to chance. More rigorous studies are required to explore the association, if any, between adrenoleukodystrophy and autoimmune diseases.

Most of the patients we reported, 83.6%, were on a continuous follow‐up within our center. This was comparable to the 79% continuous follow‐up collectively reported by five adult IMD centers in the Netherlands and Australia and the 86% reported by a center in Switzerland [1, 25]. Disease‐specific treatments were provided to 65.4% of our patients; this was below the 79% reported in the Swiss center [25]. Disease‐specific treatments in both studies included dietary measures, formulas, vitamins, cofactors, and orphan drugs. We also included chelating agents (such as for treating Wilson's disease) and stem cell transplantation (only in two patients with adrenoleukodystrophy) as disease‐specific treatments.

Approximately half of the patients had neither disability nor social support. Most patients lived in their own homes (71.2%), required no mobility aid (67.8%), and were actively working, studying, or retired due to old age (52.6%). More than a quarter of all patients fulfilled these criteria but were, in addition, fully independent, and did not require any societal support. We used strict criteria for full independence to highlight those individuals who were toward the mild end of the NIMD severity spectrum. The existence of this group of patients clearly showed that a diagnosis with NIMD does not necessarily mean debilitating disability. This underlines the need for long‐term NIMD patient outcome data to inform genetic counseling and proper management [6]. Knowledge of NIMD disease course in adults can inform optimizing their inclusion in newborn screening panels [26]. For example, early identification and dietary treatment of phenylketonuria through newborn screening are associated with favorable neurological outcomes in adulthood [27]. In contrast, delayed intervention may reduce the benefit of certain NIMD pharmacological treatments [28, 29]. However, the value of newborn screening for some NIMD remains uncertain [26]. Precision medicine and artificial intelligence can open new avenues for patients' risk stratification [30].

The limitations of our study were primarily inherent to its design as a retrospective electronic medical record‐based evaluation. We relied on ICD‐10 codes extracted from the EMR, which can be subject to misclassification and coding errors. We reviewed patients' EMR and excluded those who did not have NIMD or an established genetic diagnosis (Figure S1). Despite this review, we could not ensure the completeness of the EMR, particularly for non‐neurological symptoms and data on social support, which may have led to underestimation of certain variables. The relatively low number of individuals with NIMD in our EMR was another limitation, a limitation common in rare diseases research and highlights the need for collaborative NIMD research. The newly assigned national highly specialized care centers provide a promising collaborative platform for NIMD research in Sweden. A nationwide adult NIMD cohort can be generated by pooling data from those centers using the ICIMD classification and this study as a pilot benchmark.

In conclusion, 59.3% of adult patients with inherited neurometabolic disorders seen at Skåne University Hospital between 2020 and 2024 had disorders of intermediary energy metabolism (mainly mitochondrial disorders) or lipid metabolism (predominantly adrenoleukodystrophy). Disease‐specific treatments were provided to 65.4% of patients, 83.6% were under continuous follow‐up, and 27.1% lived a fully dependent life. More than half of the patients received no disability or social support.

## Author Contributions

B.E. and A.P. contributed to the study planning, conception, and design. B.E. collected the data. B.E., A.Y., and A.P. analyzed and interpreted the data. B.E. and A.Y. wrote the manuscript, and A.Y. provided its revised form. All authors revised the manuscript critically for important intellectual content and agreed to its submission.

## Funding

This study was funded by research grants from Region Skåne, Skåne University Hospital, the Swedish Government (via ALF, avtal för läkarutbildning och forskning) and Lund University. A.Y.'s research is supported by the Royal Physiographic Society of Lund (Fysiografiska Sällskapet i Lund) through the Nilsson‐Ehle Endowments, the Crafoord Foundation, Stiftelsen Bundy Academy, and Greta och Johan Kocks stiftelser (all in Sweden). The authors confirm independence from the sponsors; the content of the article has not been influenced by the sponsors.

## Ethics Statement

This was a retrospective review of the clinical practice, patient numbers, and population (service evaluation project), and the consent from individual patients was not sought.

## Consent

We did not seek patients' consents as the study was a service evaluation project. Data was summarized descriptively.

## Conflicts of Interest

The authors declare no conflicts of interest.

## Supporting information


**Figure S1:** Identification of patients. Patients with neurometabolic diseases were identified by searching electronic medical records and by direct information from neurologists at our center. The list of patients obtained by the two methods was assessed for overlap and misclassification. Most excluded patients did not have a neurometabolic diagnosis. However, this did not indicate misclassification as most of them had hyperlipidemia. Only patients with confirmed neurometabolic diagnosis were included. Red triangles indicate excluded patients.
**Figure S2:** Ages at onset, diagnosis, and most recent examination. Combined raincloud and box plots showing the distributions of the ages at onset, ages at diagnosis, and ages at most recent contact (examination) of the patients with the Department of Neurology, Skåne University Hospital. Circles depict the patients. The total number of patients with available ages at onset, diagnosis, and most recent examination were 55, 56, and 59, respectively. Approximately half of the patients manifested their disease before 18 years (see manuscript's text). There was an approximately 15 years difference between the median age at onset and age at most recent examination.
**Figure S3:** Diagnostic delay by diagnosis period. Box plots showing the interval between symptoms onset and receiving genetic diagnosis in each 5‐year time period. Circles depict the patients. The total number of patients with available ages at onset, diagnosis, and most recent examination were 50. Diagnosis made in most recent years was associated with longer diagnostic delays.
**Figure S4:** Clinical features. A summary of the clinical abnormalities recorded in the patients' electronic medical records is shown. We broadly classified those abnormalities to neurological and non‐neurological abnormalities.
**Table S1:** ICD‐10 codes and corresponding diagnoses. International Classification of Diseases, Tenth Revision (ICD‐10) codes used to search the electronic patients records to identify patients with inherited metabolic diseases and the corresponding metabolic diseases are shown.
**Table S2:** Swedish disability or sickness support terms explained.
**Table S3:** Disease names, classes, and inheritance and patients' sex. Diseases were classified according to the International Classification of Inherited Metabolic Disorders (ICIMD) classification. ICIMD names, common names, and online mendelian inheritance in man (OMIM) numbers were provided. AD, autosomal dominant; AR, autosomal recessive; F, female; ICIMD, International Classification of Inherited Metabolic Disorders; M, male; OMIM, online mendelian inheritance in man.

## Data Availability

Metadata is provided in the article. Individual patients’ data are not shared.

## References

[1] M. Tchan , A. Lehman , L. van Dussen , et al., “The Frequencies of Different Inborn Errors of Metabolism in Adult Metabolic Centres: 10 Years Later, Another Report From the SSIEM Adult Metabolic Physicians Group,” Journal of Inherited Metabolic Disease 48, no. 2 (2025): e70005, 10.1002/jimd.70005. 39912519

[2] B. Chandra , M. Klemmensen , B. J. Shayota , and A. L. Gropman , “From Crisis to Continuum: Redefining Survivorship in Neurometabolic Care,” Pediatric Neurology 173 (2025): 5–21, 10.1016/j.pediatrneurol.2025.08.017. 40997648

[3] M. R. Moio , J. C. Milke , Y. Moutapam‐Ngamby‐Adriaansen , et al., “Diagnosis of Inherited Metabolic Disease in Older Patients: A Systematic Literature Review,” Journal of Inherited Metabolic Disease 48, no. 3 (2025): e70038, 10.1002/jimd.70038. 40406818 PMC12100460

[4] J. M. Saudubray and F. Mochel , “The Phenotype of Adult Versus Pediatric Patients With Inborn Errors of Metabolism,” Journal of Inherited Metabolic Disease 41, no. 5 (2018): 753–756, 10.1007/s10545-018-0209-9. 29876767

[5] E. A. Ferreira , M. J. N. Buijs , R. Wijngaard , et al., “Inherited Metabolic Disorders in Adults: Systematic Review on Patient Characteristics and Diagnostic Yield of Broad Sequencing Techniques (Exome and Genome Sequencing),” Frontiers in Neurology 14 (2023): 1206106, 10.3389/fneur.2023.1206106. 37560457 PMC10408679

[6] F. Mochel , “What Can Pediatricians Learn From Adult Inherited Metabolic Diseases?,” Journal of Inherited Metabolic Disease 47, no. 5 (2024): 876–884, 10.1002/jimd.12729. 38520225

[7] E. Asimakidou , S. Pluchino , B. A. Silva , and L. Peruzzotti‐Jametti , “The Metabolic Engine of Cognition: Microglia‐Neuron Interactions in Health, Ageing and Disease,” Nature Metabolism 7 (2025): 2395–2413, 10.1038/s42255-025-01409-4. 41272201

[8] J. I. Gold , N. B. Gold , A. Strong , et al., “The Current State of Adult Metabolic Medicine in the United States: Results of a Nationwide Survey,” Genetics in Medicine 24, no. 8 (2022): 1722–1731, 10.1016/j.gim.2022.04.018. 35543711 PMC9911209

[9] A. Tummolo , G. Paterno , R. Carella , L. Melpignano , and D. De Giovanni , “Exploring Partners, Parenting and Pregnancy Thinking in Late Adolescents and Young Adults With Inherited Metabolic Disorders,” Pediatric Reports 17, no. 3 (2025): 56, 10.3390/pediatric17030056. PMC12101304 40407581

[10] J. Kido , J. Haberle , K. Sugawara , et al., “The Current Social Status in Adult Patients With Urea Cycle Disorders in Japan,” Molecular Genetics and Metabolism 145, no. 4 (2025): 109185, 10.1016/j.ymgme.2025.109185. 40618446

[11] S. Sestini , L. Paneghetti , C. Lampe , et al., “Social and Medical Needs of Rare Metabolic Patients: Results From a MetabERN Survey,” Orphanet Journal of Rare Diseases 16, no. 1 (2021): 336, 10.1186/s13023-021-01948-5. 34344397 PMC8329639

[12] J. F. Ludvigsson , D. Bergman , C. I. Lundgren , et al., “The Healthcare System in Sweden,” European Journal of Epidemiology 40, no. 5 (2025): 563–579, 10.1007/s10654-025-01226-9. 40383868 PMC12170770

[13] C. R. Ferreira , S. Rahman , M. Keller , J. Zschocke , and Group IA , “An International Classification of Inherited Metabolic Disorders (ICIMD),” Journal of Inherited Metabolic Disease 44, no. 1 (2021): 164–177, 10.1002/jimd.12348. 33340416 PMC9021760

[14] P. Jaulent , S. Charriere , F. Feillet , C. Douillard , A. Fouilhoux , and S. Thobois , “Neurological Manifestations in Adults With Phenylketonuria: New Cases and Review of the Literature,” Journal of Neurology 267, no. 2 (2020): 531–542, 10.1007/s00415-019-09608-2. 31701331

[15] M. Hult , N. Darin , U. von Dobeln , and J. E. Mansson , “Epidemiology of Lysosomal Storage Diseases in Sweden,” Acta Paediatrica 103, no. 12 (2014): 1258–1263, 10.1111/apa.12807. 25274184

[16] D. P. Germain , “Fabry Disease,” Orphanet Journal of Rare Diseases 5 (2010): 30, 10.1186/1750-1172-5-30. 21092187 PMC3009617

[17] N. Dahl , M. Lagerstrom , A. Erikson , and U. Pettersson , “Gaucher Disease Type III (Norrbottnian Type) is Caused by a Single Mutation in Exon 10 of the Glucocerebrosidase Gene,” American Journal of Human Genetics 47, no. 2 (1990): 275–278. 2378352 PMC1683716

[18] Y. S. Ng , L. A. Bindoff , G. S. Gorman , et al., “Mitochondrial Disease in Adults: Recent Advances and Future Promise,” Lancet Neurology 20, no. 7 (2021): 573–584, 10.1016/S1474-4422(21)00098-3. 34146515

[19] M. Engelen , W. J. C. van Ballegoij , E. J. Mallack , et al., “International Recommendations for the Diagnosis and Management of Patients With Adrenoleukodystrophy: A Consensus‐Based Approach,” Neurology 99, no. 21 (2022): 940–951, 10.1212/WNL.0000000000201374. 36175155 PMC9687408

[20] R. A. Sawaya , F. H. Mourad , S. Najjar , M. Mikati , and G. V. Raymond , “Adrenoleukodystrophy Associated With Vitiligo and Ulcerative Colitis,” European Neurology 42, no. 3 (1999): 169–172, 10.1159/000008093. 10529544

[21] R. Jacob , H. Mandel , and N. Shehadeh , “Guillain Barre Syndrome in a Child With X‐Linked Adrenoleukodystrophy,” Child Neurology Open 2, no. 4 (2015): 2329048X15609606, 10.1177/2329048X15609606. PMC5417021 28503596

[22] R. E. Wiersma , A. O. Gupta , T. C. Lund , et al., “Primary Adrenal Insufficiency in a Boy With Type I Diabetes: The Importance of Considering X‐Linked Adrenoleukodystrophy,” Journal of the Endocrine Society 6, no. 5 (2022): bvac039, 10.1210/jendso/bvac039. 35450414 PMC9017996

[23] A. Federico , M. T. Dotti , P. Annunziata , et al., “Adrenomyeloneurodystrophy With Late Cerebral Involvement and Evidence of a Multiple Autoimmune Disorder,” Journal of Inherited Metabolic Disease 11, no. Suppl 2 (1988): 169–172, 10.1007/BF01804227. 3141701

[24] P. Triantafyllou , M. Economou , E. Vlachaki , et al., “Multiple Endocrine Disorders Associated With Adrenomyeloneuropathy and a Novel Mutation of the ABCD1 Gene,” Pediatric Neurology 50, no. 6 (2014): 622–624, 10.1016/j.pediatrneurol.2014.01.027. 24685009

[25] K. Gariani , M. Nascimento , A. Superti‐Furga , and C. Tran , “Clouds Over IMD? Perspectives for Inherited Metabolic Diseases in Adults From a Retrospective Cohort Study in Two Swiss Adult Metabolic Clinics,” Orphanet Journal of Rare Diseases 15, no. 1 (2020): 210, 10.1186/s13023-020-01471-z. 32811506 PMC7433045

[26] M. Langeveld , S. Sirrs , D. H. Schoenmakers , et al., “Screening for Life: Perspectives From Adult Metabolic Specialists on Newborn Screening for Inherited Metabolic Diseases,” Journal of Inherited Metabolic Disease 48, no. 4 (2025): e70057, 10.1002/jimd.70057. 40610367 PMC12226250

[27] L. Aitkenhead , G. Krishna , C. Ellerton , et al., “Long‐Term Cognitive and Psychosocial Outcomes in Adults With Phenylketonuria,” Journal of Inherited Metabolic Disease 44, no. 6 (2021): 1353–1368, 10.1002/jimd.12413. 34145605

[28] C. Horgan , K. Watts , D. Ram , et al., “A Retrospective Cohort Study of Libmeldy (Atidarsagene Autotemcel) for MLD: What We Have Accomplished and What Opportunities Lie Ahead,” JIMD Reports 64, no. 5 (2023): 346–352, 10.1002/jmd2.12378. 37701322 PMC10494509

[29] C. J. Tifft , I. Batsu , R. Giugliani , et al., “Venglustat in GM2 Gangliosidoses and Related Disorders: Results of the AMETHIST Randomized Controlled and Basket Trials,” Genetics in Medicine 28, no. 1 (2026): 101615, 10.1016/j.gim.2025.101615. 41108138

[30] I. S. Forrest , H. M. T. Vy , G. Rocheleau , et al., “Machine Learning‐Based Penetrance of Genetic Variants,” Science 389, no. 6763 (2025): eadm7066, 10.1126/science.adm7066. 40875860 PMC12771675

