# Supplementary material for: Characterization of Adult Patients With Neurometabolic Disorders: A Cross‐Sectional Study at a Tertiary Neurology Center in Sweden
Source: JIMD Rep. 2026 Aug 1;67(5):e70115. doi: 10.1002/jmd2.70115 (PMC13428320; doi:10.1002/jmd2.70115)
Supplement: Supplementary file 1 — Figure S1: Identification of patients. Patients with neurometabolic diseases were identified by searching electronic medical records and by direct information from neurologists at our center. The list of patients obtained by the two methods was assessed for overlap and misclassification. Most excluded patients did not have a neurometabolic diagnosis. However, this did not indicate misclassification as most of them had hyperlipidemia. Only patients with confirmed neurometabolic diagnosis were included. Red triangles indicate excluded patients. Figure S2: Ages at onset, diagnosis, and most recent examination. Combined raincloud and box plots showing the distributions of the ages at onset, ages at diagnosis, and ages at most recent contact (examination) of the patients with the Department of Neurology, Skåne University Hospital. Circles depict the patients. The total number of patients with available ages at onset, diagnosis, and most recent examination were 55, 56, and 59, respectively. Approximately half of the patients manifested their disease before 18 years (see manuscript's text). There was an approximately 15 years difference between the median age at onset and age at most recent examination. Figure S3: Diagnostic delay by diagnosis period. Box plots showing the interval between symptoms onset and receiving genetic diagnosis in each 5‐year time period. Circles depict the patients. The total number of patients with available ages at onset, diagnosis, and most recent examination were 50. Diagnosis made in most recent years was associated with longer diagnostic delays. Figure S4: Clinical features. A summary of the clinical abnormalities recorded in the patients' electronic medical records is shown. We broadly classified those abnormalities to neurological and non‐neurological abnormalities. Table S1: ICD‐10 codes and corresponding diagnoses. International Classification of Diseases, Tenth Revision (ICD‐10) codes used to search the electronic patients records to id [file JMD2-67-e70115-s001.docx]

**Supplementary Material**

**Characterization of Adult Patients with Neurometabolic Disorders: A Cross-Sectional Study at a Tertiary Neurology Centre in Sweden**

Boel Ernerdahl^1, 2#^, Ashraf Yahia^1, 2#^, and Andreas Puschmann^1, 2, 3^

^1^Division of Neurology, Department of Clinical Sciences Lund, Lund University, Lund, Sweden.

^2^Department of Neurology, Skåne University Hospital, Lund, Sweden

^3^SciLifeLab, Lund University, Lund, Sweden.

**Outline of the Service Provided to Patients with Inherited Neurometabolic Disorders at the Department of Neurology, Skåne University Hospital**

In Sweden, 21 healthcare regions provide most primary, secondary, and tertiary care. In 2024, the Swedish National Board of Health and Welfare designated three centres in Sweden to provide national highly specialised care for inborn metabolic disorders. One of these centres is located at Skåne University Hospital in Region Skåne, southern Sweden. Children are treated within the Department of Paediatrics, adults with NIMD within the Department of Neurology, and adults with other IMD within the Department of Endocrinology. To ensure continuity of care, we have existing transition routines that support adolescents and young adults moving from paediatrics to adult services. These departments and their specialised multidisciplinary teams work in close collaboration with each other and with other clinical specialties. We also collaborate closely with the Department of Clinical Genetics in Lund, the other national highly specialized metabolic centres in Gothenburg and Stockholm, and internationally. Healthcare in Sweden is fully publicly funded.

**Supplementary Figures**

Supplementary Figure S1


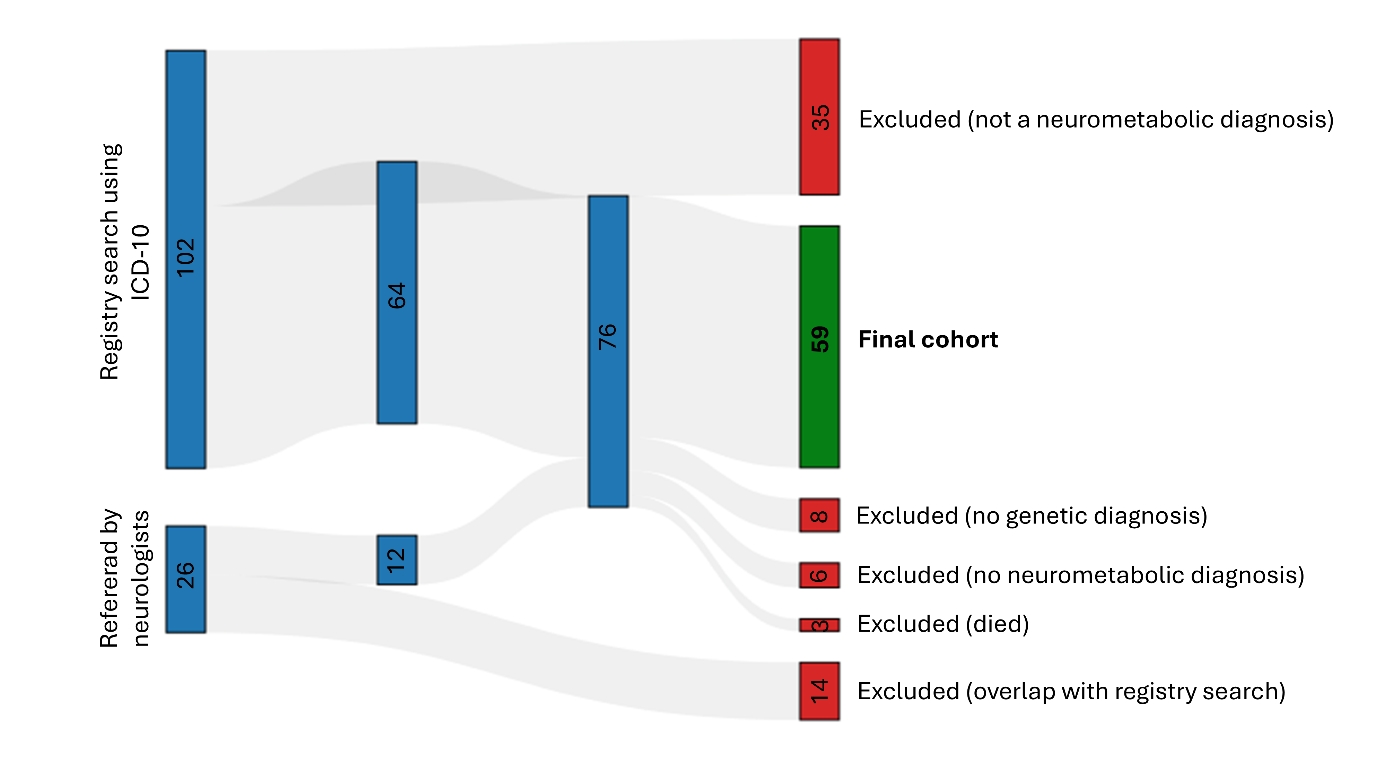


**Supplementary Figure S1**. Identification of patients. Patients with neurometabolic diseases were identified by searching electronic medical records and by direct information from neurologists at our center. The list of patients obtained by the two methods was assessed for overlap and misclassification. Most excluded patients did not have a neurometabolic diagnosis. However, this did not indicate misclassification as most of them had hyperlipidaemia. Only patients with confirmed neurometabolic diagnosis were included. Red triangles indicate excluded patients.

Supplementary Figure S2


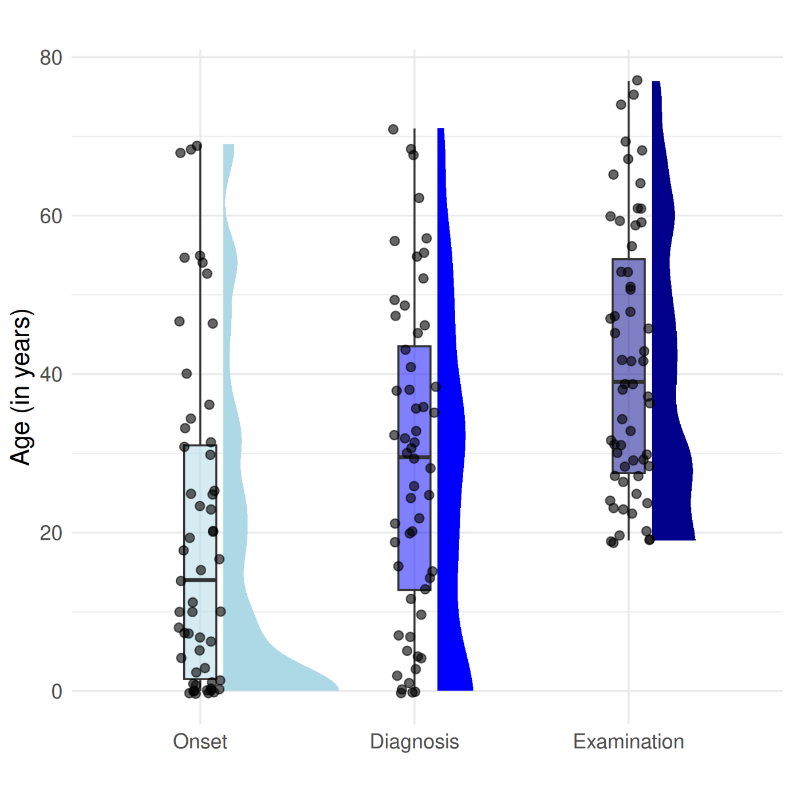


**Supplementary Figure S2**. Ages at onset, diagnosis, and most recent examination. Combined raincloud and box plots showing the distributions of the ages at onset, ages at diagnosis, and ages at most recent contact (examination) of the patients with the Department of Neurology, Skåne University Hospital. Circles depict the patients. The total number of patients with available ages at onset, diagnosis, and most recent examination were 55, 56, and 59, respectively. Approximately half of the patients manifested their disease before 18 years (see manuscript’s text). There was an approximately 15 years difference between the median age at onset and age at most recent examination.

Supplementary Figure S3


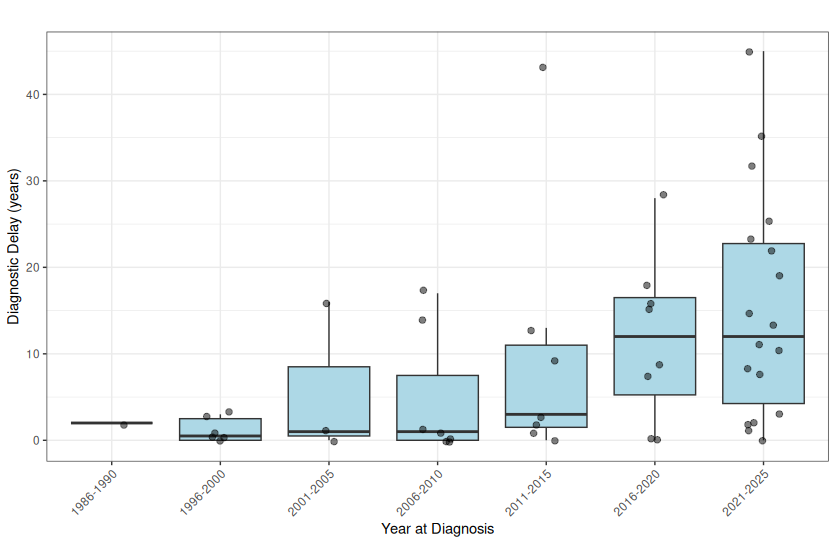


**Supplementary Figure S3**. Diagnostic delay by diagnosis period. Box plots showing the interval between symptoms onset and receiving genetic diagnosis in each 5-year time period. Circles depict the patients. The total number of patients with available ages at onset, diagnosis, and most recent examination were 50. Diagnosis made in most recent years was associated with longer diagnostic delays.

Supplementary Figure S4


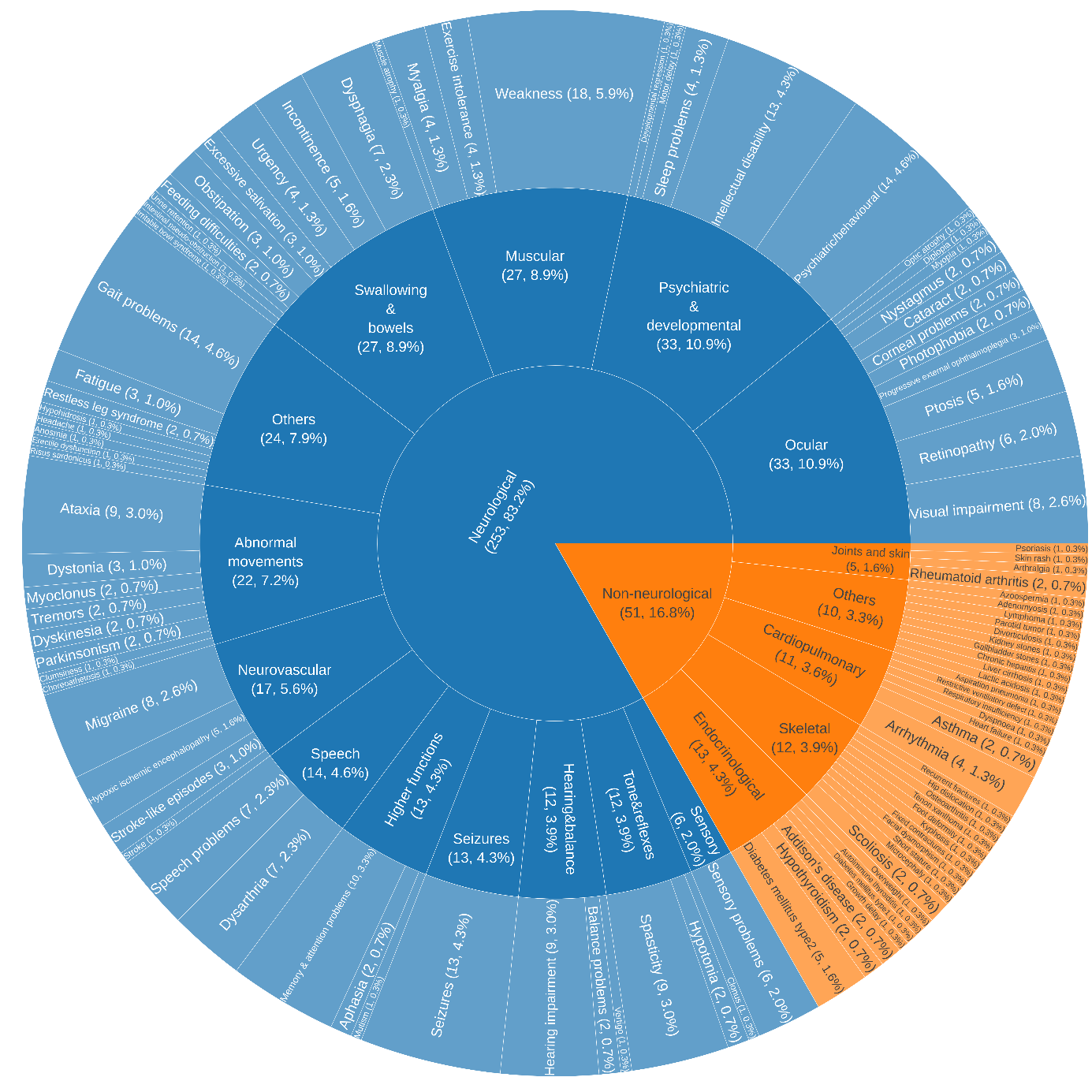


**Supplementary Figure S4**. Clinical features. A summary of the clinical abnormalities recorded in the patients’ electronic medical records is shown. We broadly classified those abnormalities to neurological and non-neurological abnormalities

**Supplementary Tables**

Supplementary Table S1

| ICD-10 code | Corresponding disease |
| --- | --- |
| E70 | Disorders of aromatic amino-acid metabolism |
| E71 | Disorders of branched-chain amino-acid metabolism and fatty-acid metabolism |
| E72 | Other disorders of amino-acid metabolism |
| E74 | Other disorders of carbohydrate metabolism |
| E75 | Disorders of sphingolipid metabolism and other lipid storage disorders |
| E76 | Disorders of glycosaminoglycan metabolism |
| E77 | Disorders of glycoprotein metabolism |
| E78 | Disorders of lipoprotein metabolism and other lipidemias |
| E79 | Disorders of purine and pyrimidine metabolism |
| E80 | Disorders of porphyrin and bilirubin metabolism |
| E83 | Disorders of mineral metabolism |
| E85.0 | Non-neuropathic heredofamilial amyloidosis |
| E85.1 | Neuropathic heredofamilial amyloidosis |
| E85.2 | Heredofamilial amyloidosis, unspecified |
| E88 | Other and unspecified metabolic disorders |
| G60.1 | Refsum's disease |
| G71.3 | Mitochondrial myopathy, not elsewhere classified |
| G73.6 | Myopathy in metabolic diseases |
| D51.1 | Vitamin B12 deficiency anemia due to selective vitamin B12 malabsorption with proteinuria |
| D51.2 | Transcobalamin II deficiency |
| **Supplementary Table S1**. ICD-10 codes and corresponding diagnoses. International Classification of Diseases, Tenth Revision (ICD-10) codes used to search the electronic patients records to identify patients with inherited metabolic diseases and the corresponding metabolic diseases are shown. | |

Supplementary Table S2

| Type of disability or sickness support from society | Swedish term | Explanation |
| --- | --- | --- |
| Personal assistance | Personlig assistans | for individuals with major and permanent disabilities to receive help with their needs from one or more persons in their everyday life. |
| Domestic services | Hemtjänst | help in an individual’s home with for example cleaning, washing, making necessary purchases but also personal care such as going to the toilet and having a shower. |
| Social care alarm | Trygghetslarm | device to call for rapid help in an individual’s home, for example after a fall. |
| Sickness compensation | Sjukersättning | a long-term economic benefit for individuals who has a disability or illness that makes them unable to work ever again |
| Activity compensation | Aktivitetsersättning | a long-term economic benefit for individuals between the age of 19 and 30 who have a disability or illness that makes them unable to work for at least one year |
| Sickness benefit | Sjukpenning | an economic benefit for sick individuals, usually for shorter periods. |
| Legal guardian | God man | a person who can help an individual with economic or legal matters, for example paying bills and applying for appropriate benefits. |
| School for Pupils with Intellectual Disabilities | Särskola | for students with an intellectual disability. |
| Accompanying person | Ledsagare | a person who accompanies an individual with a disability to for example social activities, cultural activities or hobbies |
| Housing support | Boendestöd | pedagogic, social and practical support in an individual’s home. |
| Daily work like activities | Daglig verksamhet | daily work like activities for individuals with usually an intellectual disability or autism as a substitute for paid work. |
| Nursing home | Vård- och omsorgsboende | accommodation usually for elderly people but other individuals with an extensive care need can also live there. |
| Accommodation according to Support and Service for Persons with Certain Functional  Impairments | LSS-boende | accommodation for individuals who have a major and permanent disability, usually intellectual disability but also physical ones. |
| FACT (Flexible  Assertive Community Treatment) | English term used in Swedish | home visits from psychiatry |
| Relative Caregiver Support | Anhörigstöd | offers for example counselling, information and support groups. |
| Wage subsidy | Lönebidrag | economic benefit paid to the employer to adapt the work and work environment for an employee |
| **Supplementary Table S2**. Swedish disability or sickness support terms explained. | | |

Supplementary Table S3

| International Classification of Inherited Metabolic Disorders (ICIMD) category | Disease (ICIMD name) | Other name (OMIM ID) | Gene | Inheritance | Number of patients | M | F |
| --- | --- | --- | --- | --- | --- | --- | --- |
| Disorders of lipid metabolism | Adrenoleukodystrophy | (OMIM 300100) | ABCD1 | X-linked | 10 | 4 | 6 |
|  | Sterol 27-hydroxylase deficiency | Cerebrotendinous xanthomatosis (OMIM 213700) | CYP27A1 | AR | 2 | 0 | 2 |
|  | CYP2U1 deficiency | Spastic paraplegia 56 (OMIM 615030) | CYP2U1 | AR | 1 | 1 | 0 |
|  | Phytanoyl-CoA hydroxylase deficiency | Refsum disease (OMIM 266500) | PHYH | AR | 1 | 0 | 1 |
| Disorders of amino acid metabolism | Phenylalanine hydroxylase deficiency | Phenylketonuria (OMIM 261600) | PAH | AR | 2 | 1 | 1 |
|  | Glutaryl-CoA dehydrogenase deficiency | Glutaricaciduria type1 (OMIM 231670) | GCDH | AR | 1 | 0 | 1 |
|  | Hartnup disorder | (OMIM 234500) | SLC6A19 | AR | 1 | 0 | 1 |
| Disorders of carbohydrate metabolism | Muscle glycogen phosphorylase deficiency | McArdle disease (OMIM 232600) | PYGM | AR | 1 | 1 | 0 |
| Disorders of trace elements and metals | Copper-transporting ATPase subunit beta deficiency | Wilson disease (OMIM 277900) | ATP7B | AR | 2 | 2 | 0 |
|  | Hereditary hemochromatosis type 1 | (OMIM 235200) | HFE1 | AR | 2 | 2 | 0 |
| Disorders of vitamin and cofactor metabolism | Alpha-aminoadipic semialdehyde dehydrogenase deficiency | Pyridoxine-dependent epilepsy (OMIM 266100) | ALDH7A1 | AR | 2 | 0 | 2 |
|  | 5,10-methylenetetrahydrofolate reductase deficiency | Homocystinuria due to MTHFR deficiency (OMIM 236250) | MTHFR | AR | 1 | 1 | 0 |
| Disorders of tetrapyrrole metabolism | Porphobilinogen deaminase deficiency | Acute intermittent porphyria (OMIM 176000) | HMBS | AD | 1 | 0 | 1 |
| Neurotransmitter disorders | GABA type A receptor subunit alpha 1 deficiency | Developmental and epileptic encephalopathy 19 (OMIM 615744) | GABRA1 | AD | 1 | 0 | 1 |
| Disorders of organelle biogenesis, dynamics and interactions | MSTO1 deficiency | Mitochondrial myopathy and ataxia (OMIM 617675) | MSTO1 | AR | 1 | 1 | 0 |
|  | Peroxin 16 deficiency | Zellweger syndrome (OMIM 614876) | PEX16 | AR | 1 | 1 | 0 |
|  | TANGO2 deficiency | Metabolic encephalomyopathic crises, recurrent, with rhabdomyolysis, cardiac arrhythmias, and neurodegeneration (OMIM 616878) | TANGO2 | AR | 1 | 0 | 1 |
| Disorders of complex molecule degradation | Alpha-galactosidase A deficiency | Fabry disease (OMIM 301500) | GLA | X-linked | 2 | 1 | 1 |
|  | Tripeptidyl-peptidase 1 deficiency | Neuronal ceroid lipofuscinosis type 2 (OMIM 204500) | TPP1 | AR | 1 | 1 | 0 |
|  | CLN7 disease | Neuronal ceroid lipofuscinosis type 7 (OMIM 610951) | MFSD8 | AR | 1 | 1 | 0 |
|  | AP5Z1 deficiency | Spastic paraplegia 48 (OMIM 613647) | AP5Z1 | AR | 1 | 0 | 1 |
|  | Glucocerebrosidase deficiency | Gaucher disease type 3 (OMIM 231000) | GBA1 | AR | 1 | 1 | 0 |
|  | Niemann-Pick disease type C1 | (OMIM 257220) | NPC1 | AR | 1 | 1 | 0 |
| Disorders of energy substrate metabolism | Pyruvate dehydrogenase E1 alpha deficiency | (OMIM 312170) | PDHA1 | X-linked | 4 | 1 | 3 |
| Disorders of mitochondrial DNA maintenance and replication | Mitochondrial DNA polymerase gamma catalytic subunit deficiency | _ | POLG | AR | 2 | 1 | 1 |
| Disorders of mitochondrial gene expression | Mitochondrial ribosomal small subunit 22 deficiency | Combined oxidative phosphorylation deficiency type 5 (OMIM 611719) | MRPS22 | AR | 1 | 0 | 1 |
| mtDNA-related disorders | Mitochondrial tRNA-Leu 1 deficiency | Mitochondrial myopathy, encephalopathy, lactic acidosis and stroke-like episodes; MELAS (OMIM 540000) | MT-TL1 | Mitochondrial | 8 | 2 | 6 |
|  | Mitochondrial tRNA-Asn deficiency | _ | MT-TN | Mitochondrial | 1 | 0 | 1 |
|  | Mitochondrial ATP synthase F0 subunit 6 deficiency | Neuropathy-ataxia-retinitis pigmentosa syndrome; NARP (OMIM 551500) | MT-ATP6 | Mitochondrial | 1 | 1 | 0 |
|  | Mitochondrial tRNA-Lys deficiency | Myoclonic epilepsy associated with ragged red fibers; MERRF (OMIM 545000) | MT-TK | Mitochondrial | 1 | 0 | 1 |
|  | Kearns-Sayre syndrome | Chronic progressive external ophthalmoplegia and myopathy (OMIM 530000) | _ | Mitochondrial | 2 | 1 | 1 |
| Other mitochondrial disorders | Parkin deficiency | Parkinson disease, juvenile, type 2 (OMIM 600116) | PRKN | AR | 1 | 0 | 1 |
| **Supplementary Table S3**. Disease names, classes, and inheritance and patients’ sex. Diseases were classified according to the International Classification of Inherited Metabolic Disorders (ICIMD) classification. ICIMD names, common names and online mendelian inheritance in man (OMIM) numbers were provided. ICIMD, International Classification of Inherited Metabolic Disorders; OMIM, online mendelian inheritance in man; AR, autosomal recessive; AD, autosomal dominant; M, male; F, female. | | | | | | | |
